# Low Molecular Weight Oligomers of Poly(alkylene succinate) Polyesters as Plasticizers in Poly(vinyl alcohol) Based Pharmaceutical Applications

**DOI:** 10.3390/polym13010146

**Published:** 2021-01-01

**Authors:** Artemis Palamidi, Afroditi Kapourani, Evi Christodoulou, Panagiotis A. Klonos, Konstantinos N. Kontogiannopoulos, Apostolos Kyritsis, Dimitrios N. Bikiaris, Panagiotis Barmpalexis

**Affiliations:** 1 Department of Pharmaceutical Technology, School of Pharmacy, Aristotle University of Thessaloniki, 54124 Thessaloniki, Greece; artemispalamidi@gmail.com (A.P.); akapourag@pharm.auth.gr (A.K.); kkontogi@cheng.auth.gr (K.N.K.); 2 Laboratory of Polymer Chemistry and Technology, Department of Chemistry, Aristotle University of Thessaloniki, 54124 Thessaloniki, Greece; evicius@gmail.com (E.C.); dbic@chem.auth.gr (D.N.B.); 3 Department of Physics, Zografou Campus, National Technical University of Athens, 15780 Athens, Greece; panos48al@gmail.com (P.A.K.); akyrits@central.ntua.gr (A.K.)

**Keywords:** aliphatic polyesters, biodegradable polymers, poly(vinyl alcohol), fusion-based pharmaceutical processes, thermal stability, melt flowability

## Abstract

The plasticizing effect of three low molecular weight oligomers of aliphatic poly(alkylene succinate) polyesters, namely poly(butylene succinate) (PBSu), poly(ethylene succinate) (PESu), and poly(propylene succinate) (PPSu), on partially hydrolyzed poly(vinyl alcohol) (PVA) used in melt-based pharmaceutical applications, was evaluated for the first time. Initially, the three aliphatic polyesters were prepared by the melt polycondensation process and characterized by differential scanning calorimetry (DSC), ^1^H NMR, intrinsic viscosity, and size exclusion chromatography (SEC). Subsequently, their effect on the thermophysical and physicochemical properties of PVA was thoroughly evaluated. According to the obtained results, PVA was completely miscible with all three polyesters, while PESu induced PVA’s thermal degradation, with the phenomenon starting from ~220 °C, in contrast to PBSu and PPSu, where a thermal profile similar to PVA was observed. Furthermore, molecular interactions between PVA and the prepared poly(alkylene succinate) polyesters were revealed by DSC, ATR-FTIR, and molecular dynamics simulations. Finally, melt flow index (MFI) measurements showed that, in contrast to PBSu, the use of PESu or PPSu significantly improved PVA’s melt flow properties. Hence, according to findings of the present work, only the use of low molecular weight PPSu is suitable in order to reduce processing temperature of PVA and improve its melt flow properties (plasticizing ability) without affecting its thermal decomposition.

## 1. Introduction

The poor aqueous solubility (and hence the restricted bioavailability) of the newly developed Active Pharmaceutical Ingredients (APIs) has been a major obstacle for the development of efficient drug formulations. Specifically, it is estimated that more than 50% of APIs, which are nowadays used, belong to the Biopharmaceutical Classification System class II (BCS class II), presenting poor aqueous solubility [1]. In order to overcome this problem, various approaches have been proposed, including salt formation, co-crystallization, particle size reduction, prodrug formation, use of cosolvents or surfactants, cyclodextrin complexation, etc. [2,3,4]. Out of them, amorphous solid dispersions (ASDs) were proved to be one of the most promising techniques to increase the apparent solubility and dissolution of APIs, and thus their therapeutic effect [5]. In ASDs systems, the API is amorphously dispersed within a hydrophilic (usually polymeric) matrix carrier or a mixture of carriers [6,7]. Several different approaches have been proposed for the manufacturing of ASDs, which can be categorized into two general classes: the solvent-based methods and the fusion-based methods [8]. Among them, fusion or melt -based methods such as hot-melt extrusion and melt mixing, have gained increased attention and are widely selected in the pharmaceutical arena, as they offer several advantages such as shorter time to achieve the final product and the elimination of solvent use [9,10,11].

Typical melt-based methods consist of two major subprocesses: the co-melting of the API with the carrier (or the carriers) at pre-optimized temperature, followed by the rapid cooling of the molten mixture so a one-phase system is formed [10,11]. The preparation and the physical stability of the ASDs are strongly related to the matrix-carrier’s properties, such as glass transition temperature (T_g_), hydrophilicity, plasticizing ability, etc., and API’s attributes, including glass transition temperature, logP value, hydrophilicity, etc. Furthermore, both the ratio of API to polymer/plasticizer system and the API–carrier miscibility affect the formation and the physical stability of the final system [12,13,14]. As a result, the types and grades of polymers that can be utilized in common melt-based methods, such as hot-melt extrusion (HME), are often limited by physicochemical, thermophysical, or rheological factors. Therefore, in order to improve the safety and efficacy of such drug formulations, the initial screening and assessment of the polymeric carrier must be carefully carried out [14,15].

A representative example of a polymer for such melt-based applications is polyvinyl alcohol (PVA). PVA is typically a semi-crystalline, water-soluble polymer, which is used in a wide range of industrial, commercial, medical, and food applications [16]. The physical characteristics of PVA depend on the extent or degree of its hydrolysis, specifically whether it is full or partial, which in turn dictates the classification of PVA into two groups, namely, (a) partially hydrolyzed and (b) fully hydrolyzed [17]. The degree of PVA’s hydrolysis is considered as a measure of the ratio of polyvinyl alcohol to polyvinyl acetate groups and results in PVA products of differing molecular weights, solubility, flexibility, tensile strength, and swelling-ability, which in turn can impact the performance of pharmaceutical formulations [18,19]. Its hydrophilic, non-toxic [17], non-carcinogenic and biodegradable [20] nature make PVA, and especially partially hydrolyzed PVA, an interesting polymer for pharmaceutical applications. However, the use of PVA as a matrix-carrier in melt-based pharmaceutical applications is considered a significant challenge for pharmaceutical formulation scientists, due to the fact that the polymer is not extrudable at temperatures below its melting point, whilst it is a highly thermal sensitive compound with thermal degradation taking place near its melting point [21,22].

Looking to overcome these drawbacks, recent research studies have shown that the addition of plasticizers, or lubricants, results in significant improvement of PVA’s melt flowability below its melting point [21,22,23]. In this context, a previous study of ours showed that the selection of suitable plasticizers for PVA fusion-based pharmaceutical applications is a crucial procedure and needs extreme attention when designing an efficient pharmaceutical formulation system, such as ASDs [24]. Based on these findings, the study concludes that there is still a lot of ground to be covered in this area, due to the endless urge for new and efficient plasticizers, able to reduce PVA’s processing temperature, improve its melt flow characteristics, and maintain its acceptable thermal stability.

On this set framework, the scope of the present study was to evaluate, for the first time, the use of three poly(alkylene succinate) polyesters as suitable pharmaceutical plasticizers in melt-based pharmaceutical applications utilizing partially hydrolyzed PVA as a matrix carrier (such as the formation of PVA-based ASDs). Generally, biodegradable polymers have attracted considerable attention last decades due to their potential applications as green materials, contributing to lower environmental pollution. The examined three aliphatic polyesters present favorable features of controllable biodegradation rate, biocompatibility, and high processability [25,26]. Hence, they are appropriate for medical and biomedical applications, including drug delivery systems. In the present study, the effect of poly(ethylene succinate) (PESu), poly(propylene succinate) (PPSu), and poly(butylene succinate) (PBSu) on the thermo-physical and physicochemical properties of PVA was evaluated via differential scanning calorimetry (DSC), thermogravimetric analysis (TGA), hot stage polarized light microscopy (HSM), powder X-ray diffraction (pXRD), and attenuated total reflectance (ATR) FTIR analyses. In addition, the plasticizing ability of the prepared polyesters on the melt-flow properties of PVA was also evaluated, while molecular dynamics (MD) simulations were employed for the in-depth understanding of the physicochemical phenomena occurring during the co-melting process of PVA with the selected polyesters.

## 2. Materials and Methods

### 2.1. Materials

Partially hydrolyzed PVA (Parteck^®^ MXP, lot no. F2016164812) with 87–89% hydrolysis grade and MW approx. 32,000 Da, was obtained from Merck Millipore (Merck Millipore, Burlington, Massachusetts, United States). Succinic acid (SA) (purum 99%+), ethylene glycol (anhydrous, 99.8%) used in the synthesis of polyesters were purchased from Sigma-Aldrich, 1,3-propanediol (purum 99.6 + %), and 1,4-Butanediol (assay 99%) were purchased from Alfa Aeser, (Kandel, Germany). Antimony trioxide (Sb_2_O_3_, 99.99%), used as catalyst, was of analytical grade, and it was purchased from Aldrich Co. (Chemie GmbH, Steinheim, Germany). All other reagents were of analytical or pharmaceutical grade and used as received.

### 2.2. Synthesis of Poly(alkylene succinate) Polyesters

**Figure 1:**
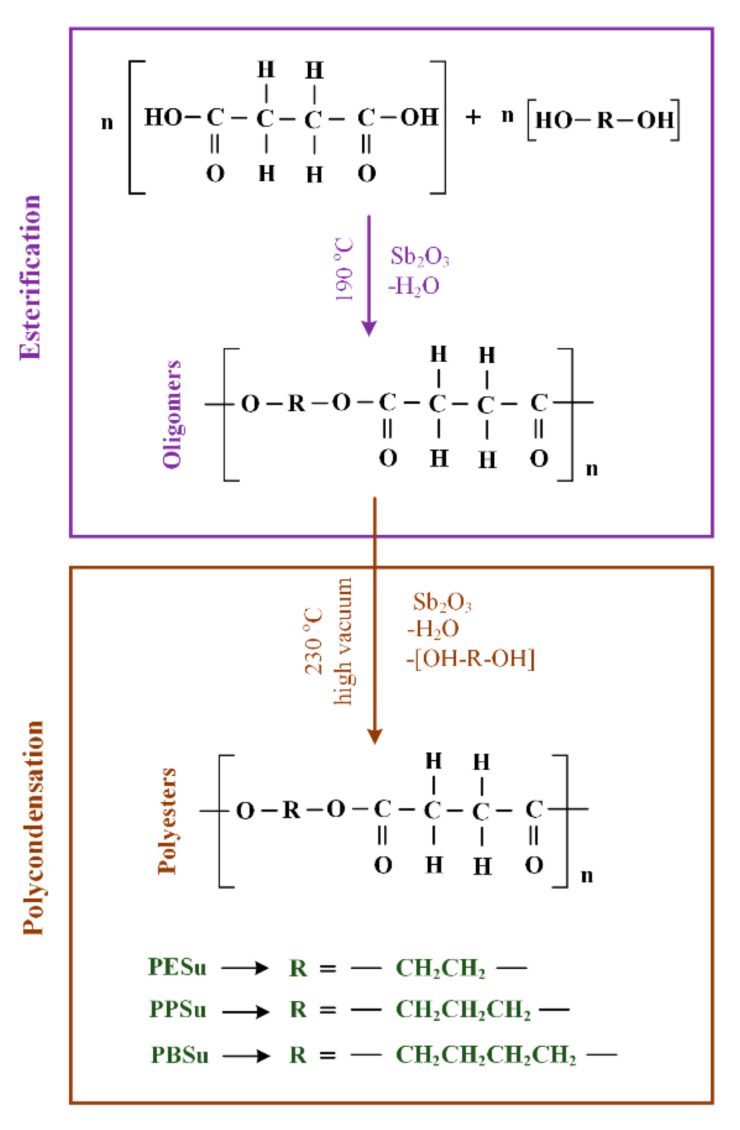
Chemical reaction scheme for the preparation of the poly(alkylene succinate) polyesters via the two-stage melt polycondensation method (esterification-polycondensation).

Synthesis of aliphatic polyesters was performed following the two-stage melt polycondensation method (esterification and polycondensation) in a glass batch reactor [27,28]. The reaction scheme is shown in Figure 1.

In brief, the proper amount of succinic acid and appropriate diol, in a molar ratio 1/1.1, and the catalyst Sb_2_O_3_ (400 ppm relative to succinic acid) were charged into the round bottom flask of the polycondensation apparatus. The apparatus with the reagents was evacuated several times and filled with nitrogen (N_2_) in order to remove oxygen. During the first reaction step (esterification), reagents were heated at 180 °C for 4 h under 500 rpm stirring and N_2_ flow (50 mL/min). Water was distilled (as reaction by-product) and was collected in a graduated cylinder. When almost all the theoretical amount of H_2_O was collected (after 3.5 h), the first step was considered as completed. Thereafter, in the second step of polycondensation, temperature was gradually increased from 180 to 230 °C (15 min) and vacuum was slowly implemented (5.0 Pa) in order to remove unreacted monomers, avoid excessive foaming, and minimize oligomer sublimation. The polycondensation continued for about 15 min, at 230 °C, for all prepared polyesters, while stirring speed was also increased to 750 rpm. After that time, polymerization was terminated by rapid cooling to room temperature and the polyesters were removed. It is important to note that in the present study the catalyst (Sb_2_O_3_) was not removed from the prepared polyesters, since its concentration was extremely low (400 ppm relative to succinic acid), and hence no effect on the physicochemical or thermophysical properties of the prepared polyesters is anticipated. However, before proceeding to an in vivo evaluation (in following studies), a purification method should be adopted in order to remove the catalyst since there are several reports raising serious concerns on its toxicity. The prepared polyesters were stored in hermetically sealed glass vials and placed in desiccators (25 °C) before further use.

### 2.3. Characterization of Poly(alkylene succinate) Polyesters

#### 2.3.1. Intrinsic Viscosity

The intrinsic viscosity [*η*] measurements of the obtained polyesters were performed by using an Ubbelohde viscometer (Schott Gerate GMBH, Hofheim, Germany). The samples were dissolved in chloroform (CHCl_3_) at room temperature in order to prepare solutions of 1 % *w*/*v*, and filtered through a disposable membrane filter 0.2 μm (Teflon). Intrinsic viscosity was calculated after the Solomon-Ciuta equation [29]:

(1) $$\left[\eta \right]=\frac{{\left[2\left\{t/t_{0}-\ln\left(t/t_{0}\right)-1\right\}\right]}^{1/2}}{c}$$

where, *c* is the concentration of the solution, *t* is the flow time of solution and *t*_o_ represents the flow time of pure solvent. The number-average molecular weight (${\bar{Μ}}_{n}$) of the samples was calculated from intrinsic viscosity [*η*] values, using the Berkowitz equation:

(2) $${\bar{Μ}}_{n}=3.29\times 10^{4}{\left[\eta \right]}^{1.54}$$

#### 2.3.2. Size Exclusion Chromatography (SEC)

SEC was used for the determination of the molecular weight distribution and the average molecular weights of the samples. Molecular weight determinations were performed using a high temperature SEC system by Waters (model 600) equipped with a refractive index detector (Milford, MA, USA) and Water Styragels columns (Milford, MA, USA) in the order of HR1, HR2, HR4, HR4, and HR5. All samples were dissolved in CHCl_3_ at a constant concentration of 12 mg/600 μL and filtrated. The elution solvent was CHCl_3_ at a constant flow rate of 1 mL/min and the measurements were performed at 35 °C and 150 μL injection volume. Calibration was performed using nine polystyrene standards with narrow molecular weight distribution, 1000–300,000 molecular weight distribution.

#### 2.3.3. Nuclear Magnetic Resonance Spectroscopy (^1^H-NMR)

^1^H-NMR spectra of polyesters (5% *w*/*v*) were recorded in deuterated chloroform (CDCl_3_), on an Agilent 500 spectrometer (Agilent Technologies, Santa Clara, CA, USA), at room temperature. The number of scans was 16 and the sweep width was 6 kHz.

#### 2.3.4. DSC Measurements

For the determination of polyesters’ thermophysical properties, DSC studies were performed by means of a TA Q200 series DSC instrument (TA, USA). Each sample was scanned using a cyclic scanning procedure. Specifically, the samples were heated from 20 to 190 °C at a heating rate of 10 °C /min and were maintained at that temperature for 3 min in order to achieve equilibrium and erase any thermal history. Then, the samples were quench cooled at −100 °C, kept at that temperature for 3 min and heated again up to 190 °C with 10 °C /min. The melting point (T_melt_) and the crystallization temperature (T_cryst_) were determined as the onset temperature of the corresponding heat flow peak/event. The T_g_ was determined as the inflection point temperature of the heat flow curve, while the enthalpy of fusion (ΔH_f_) and crystallization (ΔH_c_) were determined as the integrated area of the total heat flow curve.

### 2.4. Preparation of PVA-Polyesters Melt Mixtures

PVA-polyester dispersions were prepared by melt-quenching approach at ratios of 90/10, 80/20, and 70/30 *w/w* of PVA to aliphatic polyester. Briefly, appropriate amounts of PVA were mixed with polyesters in a mortar and pestle, placed in appropriate aluminum pans and then heated at 215 °C for 5 min until a homogeneous melt dispersion (verified visually and via polarized light microscopy) was obtained. The dispersion was then rapidly cooled in an ice bath, without temperature control, and subsequently pulverized at room temperature with mortar and pestle, and then sieved from a 300 μm sieve. All samples were placed in hermetically sealed amber glass vials and stored in a desiccator at room temperature (25 °C) in order to prevent moisture absorption before further analysis.

### 2.5. Miscibility Evaluation

#### 2.5.1. Theoretical Evaluation

Hansen solubility parameters (HSP) calculated based on the Hoftyzer-Van Krevelen (HVK) group contribution method, were used in order to theoretically evaluate the miscibility of PVA with the prepared polyesters. Based on this approach, the total solubility parameter (*δ_t_*) of a molecule describes its ability to interact with other molecules based on the contributions of molar volume (*V*), dispersion (*F_d_*), polar (*F_p_*) and hydrogen bonding (*E_h_*) forces according to the following equations [30]:

(3) $$\delta_{t}=\sqrt{\delta_{d}^{2}+\delta_{p}^{2}+\delta_{h}^{2}}$$

(4) $$\delta_{d}=\frac{\sum_{i}F_{di}}{\sum_{i}V_{i}},\;\delta_{p}=\frac{\sqrt{\sum_{i}F_{pi}^{2}}}{\sum_{i}V_{i}},\;\delta_{h}=\frac{\sqrt{\sum_{i}E_{h}}}{\sqrt{\sum_{i}V_{i}}}$$

where, *δ_d_*, *δ_p_*, and *δ_h_* are the partial solubility parameters for intermolecular dispersion, polar, and hydrogen bonding forces, respectively.

In addition to HSP, the miscibility of compounds was also evaluated theoretically via MD simulations. Using this approach, the miscibility of compounds is evaluated in the melt, instead of 25 °C, which is crucial in melt-based pharmaceutical processes. Details on the methodology followed for MD simulations are given in Section 2.9.

#### 2.5.2. Experimental Evaluation

Hot stage polarized light microscopy (HSM) was used for the experimental verification of a component’s miscibility during melting. Specifically, physical mixtures of PVA and polyesters (at ratio of 90/10, 80/20, and 70/30 *w/w* PVA to polyester) were heated from 25 °C until complete PVA melting (~210 °C), using a rate of 10 °C /min on a Linkam THMS600 heating stage (Linkam Scientific Instruments Ltd., Surrey, UK), mounted on Olympus BX41 polarized light microscope, and controlled through a Linkam TP94 temperature controller. Evaluation of miscibility was made by visual observation.

### 2.6. Thermal Properties Evaluation

DSC pans containing accurately weighted amounts (~5.0 mg) of samples (PVA-polyesters mixtures) were melt-quenched by heating up to 230 °C with a heating rate of 10 °C /min, keeping it isothermal for 3 min, in order to erase any thermal history, cooling at a rate of 10 °C /min to 20 °C, before re-heating again at the same rate up to approx. 10 °C above PVA’s melting point. The instrument was calibrated for temperature using benzophenone, indium, and tin. The enthalpic response was calibrated using high purity indium. The melting point temperature (T_m_) was determined as the onset temperature of the heat flow curve, the glass transition temperature (T_g_) was determined as the inflection point temperature of the heat flow curve, while the enthalpy of fusion (ΔH_f_) was determined as the integrated area of the total heat flow curve. The standard deviations of temperatures and enthalpies determined in this work were not higher than 1.0 °C and 3.0 J/g, respectively. All experiments were conducted in triplicate.

Thermo-gravimetric analysis (TGA) was performed in a Shimadzu TGA-50 thermogravimetric analyzer (Tokyo, Japan). Approximately, 5.0 mg of samples (pure components and PVA-polyesters mixtures at 90/10, 80/20, 70/30 % *w/w* PVA to polyester ratio) were placed into an aluminum sample pan, which was attached to a sensitive microbalance assembly, and heated from 25 to 300 °C at a rate of 10 °C /min, using nitrogen as purge gas at a flow rate of 25 mL/min. All experiments were performed in triplicate.

### 2.7. Physical State Evaluation

Powder X-ray diffracrometry (pXRD) patterns of the pure components (PVA and polyesters) and the PVA-polyesters melt mixes (prepared according to Section 2.4) were recorded using an X-ray diffractometer (Rigaku—Miniflex II) with a CuKα radiation source for crystalline phase identification (λ = 0.15405 nm for CuKα). All samples were scanned from 5 to 50° 2 <theta>, at steps of 0.05°.

### 2.8. Molecular Interactions Evaluation

#### 2.8.1. Experimental Evaluation

Molecular interactions between PVA and the tested polyesters were experimentally studied via attenuated total reflectance FTIR spectroscopy (ATR-FTIR). Specifically, the ATR-FTIR spectra of pure components, physical mixtures, and melt mixtures were obtained in the region of 750–4000 cm^–1^ using a Shimadzu IR-Prestige-21 FT-IR spectrometer (Tokyo, Japan), coupled with a horizontal Golden Gate MKII single-reflection ATR system (Specac, Kent, UK) equipped with ZnSe lenses after appropriate background subtraction. Sixty-four (64) scans over the selected wavenumber range at a resolution of 4 cm^−1^ were averaged for each sample.

#### 2.8.2. Theoretical Evaluation

In order to gain an in-depth understanding of the molecular interactions evolving between PVA and the studied polyesters, MD simulations were conducted (details on the methodology followed are given in the following section).

### 2.9. Molecular Dynamics (MD) Simulations

#### 2.9.1. Preparation of Initial Structures

The initial structure of PVA (50 monomers) having a 90% degree of hydrolysis (five hydroxyl units were replaced with acetyl units) was prepared based on a previous study of ours [24]. The initial structures of PPSu, PESu and PBSu (having five monomers each) were constructed using VEGAZZ v.3.1.2.39 [31]. All molecular structures were then optimized via energy minimization simulations runs using the pcff_d force field [32] with XenoView v.3.7.9.0 suite [33]. During geometry optimization, tolerance was set at 1.00E-4 kcal/mol, while maximum displacement per iteration was set at 10 Å. A total of ten structures (for each component) were optimized and those with the lowest energy were selected for further analysis.

#### 2.9.2. Contraction of Amorphous Cells

In order to build the amorphous cells, Xenoview’s amorphous builder was used by varying the rotatable torsions via the free rotation model method. A total of ten independent simulation boxes were constructed having twenty PPSu, PESu and PBSu chains and minimized steepest descent algorithm with 1.00 × 10^−4^ kcal/mol tolerance and a 10 Å maximum displacement per iteration in order to remove unfavorable interactions and attain the lowest energy state. The amorphous cell of PVA was obtained from a previous study of ours [24]. The structure with the lowest energy was selected for further processing. The cell structure having the lowest energy in each case was further equilibrated following a multistep equilibration protocol proposed by Li et al. [34], during which the torsion angles have sufficient time to relax into new distributions under the effect of full non-bonded interactions. In this way, the chain conformation differences within the initial chain growth step are being eliminated.

#### 2.9.3. MD Simulation Runs

A two-phase MD simulations protocol was adopted. Initially, an equilibration phase was employed in order to relax the obtained structures for 2.0 ns under the NVT ensemble at 200 °C (approximately the melting temperature of PVA) with pcff_d force field, 1.0 fs time step and Berendsen thermostat. Then in the production phase the structures obtained were subjected in a further 1.0 ns processing under NPT (variable volume and shape) at atmospheric pressure (1 atm) and 200 °C, using a cut-off radius of 10.0 Å, spline distance of 1 Å, Berendsen thermost, Berendsen barostat, and 1.0 fs time step. In all cases, the validity of the simulations was ensured by following the criteria suggested by van Gunsteren and Mark [35].

Determination of compound’s miscibility: In order to evaluate the miscibility of components, the assemblies obtained from the previous MD runs were subjected in a further 200 ps processing under NPT with the same MD parameters. The final 50 ps of the trajectory were used for computing the cohesive energy (*E_coh_*) and the solubility parameter (*δ*) according to the following equation:

(5) $$\delta =\;\sqrt{\frac{{Ε}_{coh}}{V}}$$

where, *V* is the volume estimated by MD simulations.

*Interaction energy (E_inter_)*: E_inter_ is a characteristic parameter which can represent the interaction force of components in composite system. The interaction energy can be evaluated by the total energy of the blends and each component in the system according to the following equation [36]:

E_inter_ = E_tot_(*PVA/polyester*) − E_tot_(*PVA*) − E_tot_(*polyester*) (6)

where, E_tot_(*PVA/plasticizer*) is the energy of the polymer/polyester system, E_tot_(*PVA*) is the energy of pure PVA and E_tot_(*polyester*) is the energy of the pure poly(alkylene succinate) polyesters.

*Molecular interactions*: The radial distribution function, RDF or g(r), was calculated for PVA’s hydroxyl hydrogens around the poly(alkylene succinate) polyesters’s oxygens at 200 °C in order to gain an insight into the molecular interactions evolving between the compounds during the melting process.

### 2.10. Melt Flow Index (MFI)

The MFΙs of pure PVA and PVA- poly(alkylene succinate) polyester melts (at a ratio of 30% *w/w* polyester to PVA) were measured at temperatures 190, 200, 210, and 220 °C, using CEAST’s Melt Flow Quick Index meter (CEAST, Turin, Italy) according to the ASTM standard D 1238-04 and ISO standard 1133 (load 2.16 kg).

## 3. Results and Discussion

### 3.1. Characterization of Poly(alkylene succinate) Polyesters

The proposed two-stage polycondensation method resulted in the formation of poly(alkylene succinate) polyester having an almost white (PESu and PBSu) to light-brown (PPSu) color. All prepared materials were soft (waxy-like) with PESu being more brittle and PPSu being more elastic than the others.

#### 3.1.1. H NMR Results

**Figure 2:**
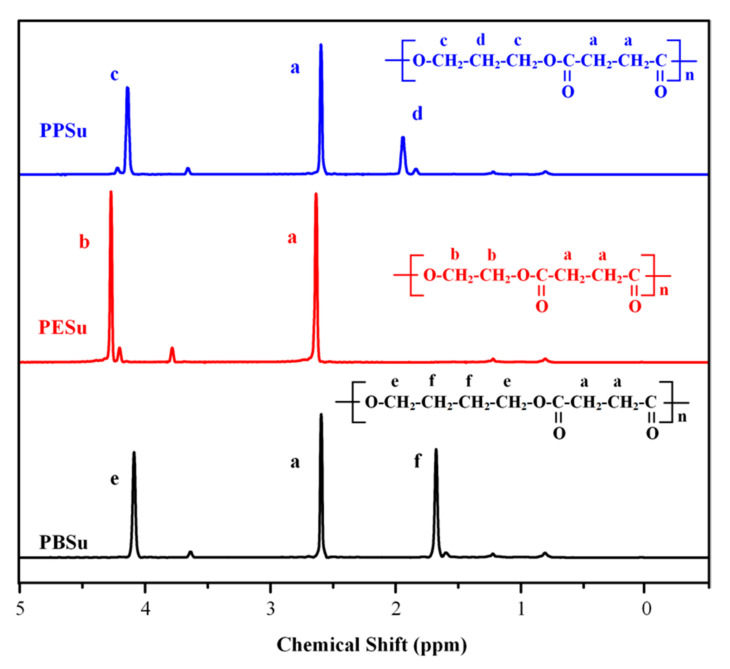
^1^H-NMR spectra of the prepared poly(alkylene succinate) polyesters (PPSu, PESu and PBSu).

The obtained ^1^H NMR spectra for the three polyesters are presented in Figure 2. The ^1^H NMR spectrum of PESu showed only two characteristic peaks at 2.55–2.67 and 4.18–4.3 ppm attributed to methylene protons of succinic acid, a, and of ethylene glycol, b, respectively. In the case of PPSu, there was a single peak at 2.63 ppm attributed to methylene protons of succinic acid, a, a triple peak at 4.09–4.21 ppm attributed to c protons and a multiple peak between 1.9 and 2.02 ppm corresponding to d protons. PBSu ^1^H NMR spectrum presented a triple peak at 4–4.2 ppm attributed to e proton groups and a multiple peak at 1.8–2 ppm attributed to f proton groups. The above- mentioned results were in agreement with previous studies [37,38,39], indicating that the selected two-stage melt polycondensation method (i.e., esterification and polycondensation) was able to prepare the desired poly(alkylene succinate) polyesters (i.e., PESu, PPSU, and PBSu).

#### 3.1.2. Intrinsic Viscosity—Molecular Weight

Table 1 summarizes the intrinsic viscosity and SEC results for the three prepared polyesters. PPSu showed the lowest intrinsic viscosity (with a value of 0.07 dL/g) which was significantly lower compared to the rest polyesters (0.13 and 0.16 dL/g for PESu and PBSu, respectively). Gel permeation chromatography measurements showed that all polyesters had relatively low average molecular weights (Mw) with values ranging from 3056 g/mol for PESu to 6551 and 8833 g/mol for PPSu and PBSu, respectively. Polydispersity (i.e., Mw/Mn) ranged from 2.08 to 2.56 which is typical for such polyesters [37].

#### 3.1.3. Thermal Properties

**Figure 3:**
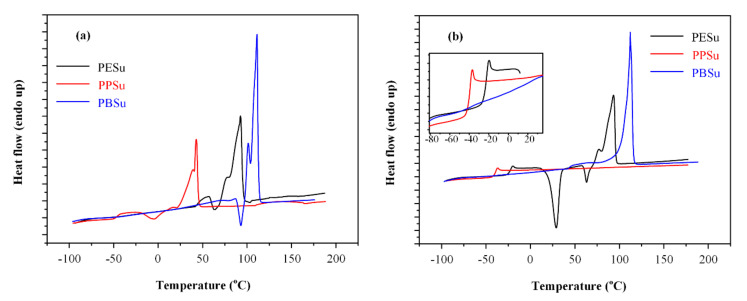
DSC thermograms of polyesters (**a**) as received from glass reactor and (**b**) after quenching cooling.

Since the aim of the present study was to evaluate the plasticizing ability of poly(alkylene succinate) polyesters in melt-based pharmaceutical applications (such as ASDs utilizing PVA as a suitable matrix carrier), polyesters’ thermal properties were analyzed via DSC. Figure 3a presents the 1st heating DSC scan thermograms for the pure polyesters, while Table 1 summarizes the observed thermal events. Results showed that PPSu exhibits a significantly lower melting temperature (T_m_ = 45 °C) compared to PBSu and PESu (T_m_ at 111 and 93 °C, respectively), while all polyesters showed a small recrystallization exotherm prior to melting, indicating that the prepared components were semi-crystalline in nature. After melting, all polyesters were subjected to rapid quench cooling in order to obtain completely amorphous samples. Figure 3b shows the DSC heating thermograms of the samples after melt-quench cooling. According to the obtained results, PPSu and PBSu presented similar T_g_ values (at −40 °C and −41 °C, respectively), while PESu showed a slightly higher T_g_ (at −24 °C). In addition, results showed that PPSu was the only polyester that remained completely amorphous (at least within the experimental time scale for scans at 10 °C/min). This is extremely important when the use of plasticizer is intended for pharmaceutical applications targeting the complete drug amorphization (such as hot-melt extrusion), since the crystallinity of the matrix compounds (i.e., the polymer and plasticizer) may act as a substrate for drug’s recrystallization. Finally, the crystalline fraction (CF_c_) for the rest polyesters (Table 1), calculated based on the heat of fusion for pure crystalline materials (i.e., 180 J/g for PESu, and 210 J/g for PBSu) [26], showed significantly less recrystallization for PESu compared to PBSu (5 compared to 30 wt %, respectively), indicating that PESu is less susceptible to recrystallization compared to PBSu.

### 3.2. PVA- Poly(alkylene succinate) Polyesters Characterization

#### 3.2.1. Miscibility Evaluation

In general, plasticizers reduce the melting temperature and glass transition temperatures (T_g_) of polymers (such as PVA) via a process called plasticization (described by the free volume theory). However, in order for this to happen, the two components (i.e., the polymer and the plasticizer) must be completely miscible with each other. In addition, components’ miscibility (referring to the ability of producing a homogeneous single phase where the components are mixed at a molecular level) significantly affects the physical stability of the drug-polymer-plasticizer system [40]. An immiscible polymer-plasticizer system could lead to unexpected destabilization, which in turn results to a higher tendency for the API to recrystallize (i.e., change in its physical state) and subsequently to a change in its bioavailability profile [41,42].

##### Theoretical Evaluation

HSP method: Initially, the miscibility of PVA with the three prepared polyesters was evaluated based on the difference of the HSPs (Δ*δ*_t_) of components estimated via the HVK group contribution method. This approach is based on Greenhalgh et al. suggestion that components are immiscible when *Δδ_t_* > 10 MPa^1/2^, miscible when Δ*δ*_t_ < 7 MPa^1/2^ and likely to form a glassy solid solution when Δ*δ_t_* < 2 MPa^1/2^ [43]. Additionally, systems with a difference of 7.4 to 15 MPa^1/2^ are considered slightly immiscible in the liquid state, while total immiscibility is observed in systems with differences greater than 15 MPa^1/2^ [44]. Based on the above, the HSP, along with the Δ*δ*_t_ (in absolute values) for all systems are summarized in Table 2. Results showed that, at least according to the HSPs, the studied polyesters are slightly immiscible with PVA in the liquid state, since the absolute difference in HSP values were between 7.4 and 15.0 MPA^1/2^ (namely 12.4, 12.1 and 11.9 MPA^1/2^ for PESu, PPSu and PBSu, respectively). However, although widely employed, the evaluation of miscibility via HSPs via the HVK method presents several limitations, including the fact that the estimations do not account for the effect of temperature or the molecular interactions evolving between the systems’ components, such as hydrogen-bonds [44].

**Figure 4:**
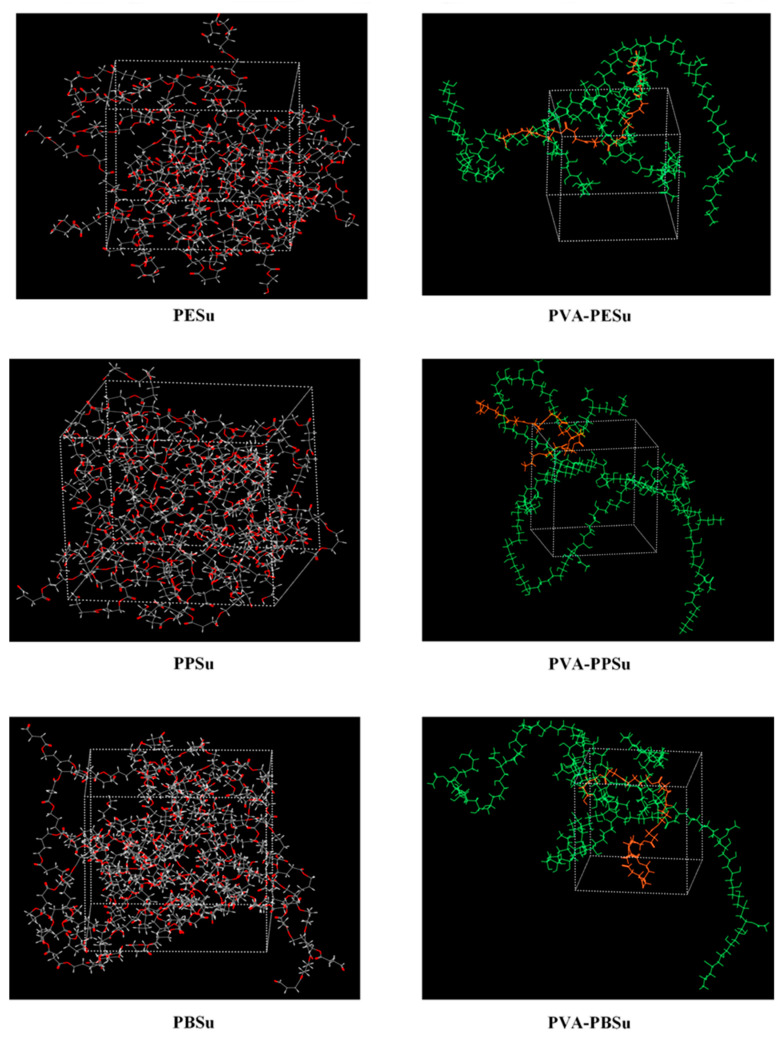
MD-simulation boxes containing twenty polyester chains (hydrogen atoms with white, carbon atoms with grey and oxygen atoms with red) or the mixtures of the polyesters (orange) with PVA (green).

MD simulations: In order to overcome some of the limitations associated with the HSP calculations based on the group contribution method, the miscibility of components was also evaluated theoretically via MD simulations. In this approach, the solubility parameters of components (*δ_MD_*) were estimated at 200 °C based on Equation (5). Figure 4 shows the MD-simulation boxes containing the 20 polyester chains used to evaluate the solubility parameter *δ_MD_*. In the case of PVA, the results were obtained from a previous study of ours [24]. The MD-based solubility parameters along with the Δ*δ*_t_ results are presented in Table 2. Results showed that in all cases, at least based on MD simulations, the prepared poly(alkylene succinate) polyesters may be considered as completely miscible with PVA, since all estimated *δ_MD_* differences were below 2.0. This result is not in agreement with the previous suggestions made by HSPs analysis, where the two components were identified as being slightly immiscible. The differences observed between the two approaches (i.e., the MD simulations and the group contribution method) are probably due the fact that the group contribution method depends on constant group values that do not account for the thermodynamic and kinetic state of the compounds as well as the fact that the latter case does not account for the effect of the temperature [45].

##### Experimental Evaluation

**Figure 5:**
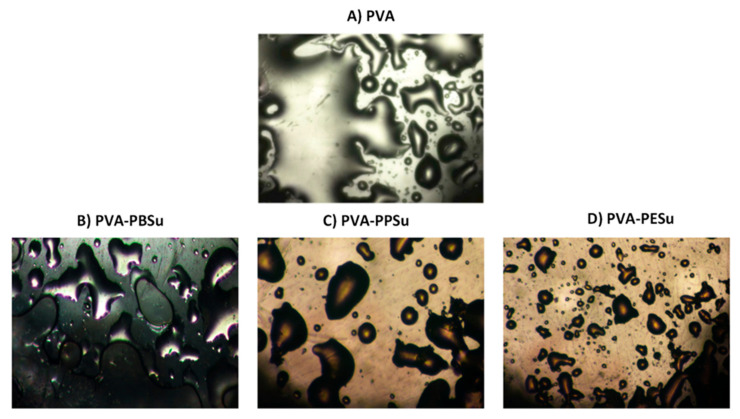
HSM photographs for the melting processes of (**A**) pure PVA, (**B**) PVA-PBSu, (**C**) PVA-PPSu and (**D**) PVA-PESu at 210 °C.

The most frequently used experimental technique to prove components’ miscibility is by far the identification of a single T_g_ using DSC [42,46]. However, according to recent studies, the evaluation of miscibility via DSC is related to several drawbacks and limitations, leading to inadequate results [46,47]. Among the several other alternative techniques suggested (such as pXRD and solid-state NMR) HSM may be considered as the easiest to implement. With HSM, miscibility in the melt state can be easily detected, since miscible compounds form a uniform melt region, while the interfacial tensions in immiscible compounds cause each melt to maintain their surface as small as possible, forming thus an ‘oil-water’ like immiscible melt region [24]. Figure 5 presents the HSM obtained images at the melting point of the pure PVA and the PVA-polyesters systems. Based on the obtained results, PVA and the examined polyesters were completely miscible at the melt state, since no de-mixing zones were observed between the components. This result indicates that the use of MD-simulations is more suitable to predict the melt miscibility of components compared to the HSP approach using the group contribution method.

#### 3.2.2. Evaluation of Thermal Properties

##### TGA Results

**Figure 6:**
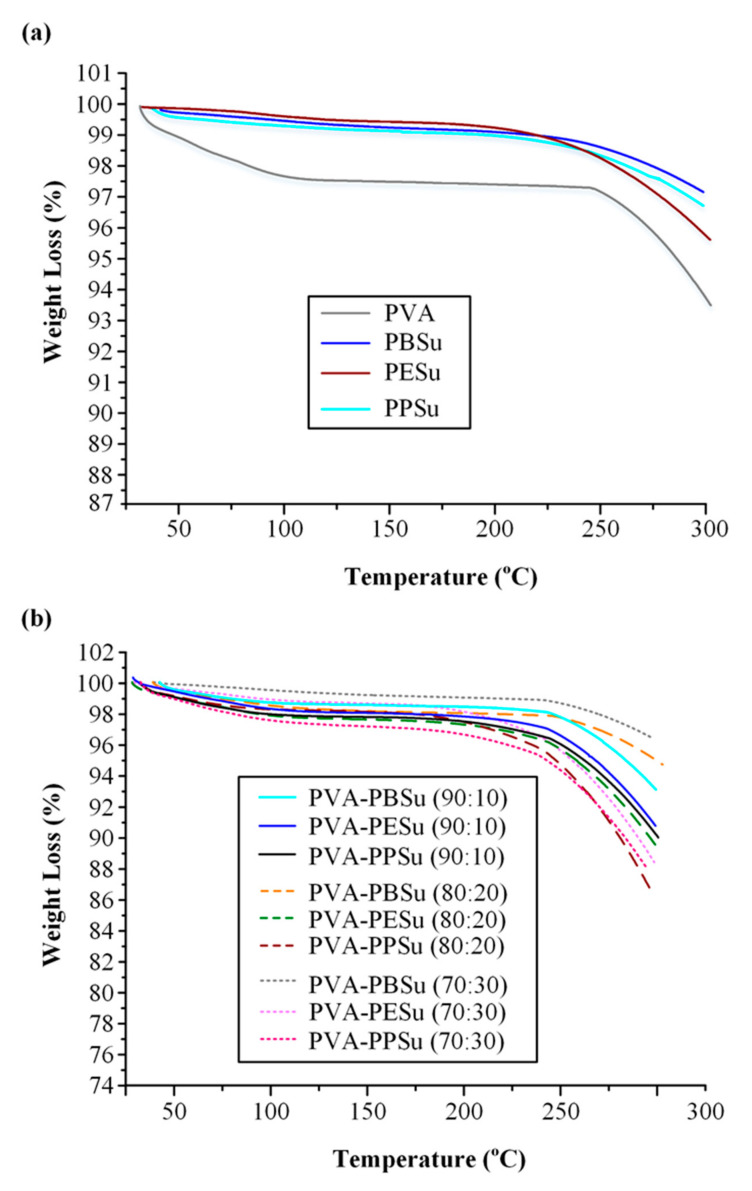
TGA thermograms of (**a**) neat PVA, PBSu, PESu and PPSu and (**b**) PVA-polyester mixtures at several weight ratios.

As stated in the introduction section, one of the most important difficulties in PVA’s utilization as a matrix-carrier in melt-based pharmaceutical applications (such as hot-melt extrusion) is its high thermal sensitivity (with thermal degradation taking place near its melting point). Therefore, when selecting an appropriate plasticizer, it is important to ensure that its use does not worsen PVA’s thermal degradation profile. Hence, in order to evaluate the effect of the prepared poly(alkylene succinate) polyesters on the thermal degradation of PVA, TGA analysis was performed. Figure 6a shows the TGA thermograms for pure PVA, PBSu, PESu and PPSu, while Figure 6b presents the TGA thermograms for PVA-polyesters mixtures at several PVA to polyester ratios (i.e., 90/10, 80/20 and 70/30 *w/w* PVA to polyester). In regard to pure components, results showed that PVA is thermally stable up to approximately 250 °C, while it presented only a small weight loss (~2 % *w/w*) at temperatures below 100 °C (indicative of the residual moisture water loss). PBSu and PPSu showed a similar thermal degradation path, while PESu was thermally stable up to approximately 220 °C. As far as the PVA-PBSu and PVA-PPSu PMs are concerned, the obtained results illustrate a similar thermal behavior to pure PVA for all weight ratios studied (i.e., thermal degradation at ~250 °C), while PESu seems to induce PVA’s thermal degradation (with the weight loss starting at lower temperatures). Specifically, in the case of PESu, the increase of the polyester content results into the formation of more thermally unstable systems, with PVA’s degradation taking place at ~220 °C when 30 wt %. of the polyester is used in the mixture.

##### DSC Results

**Figure 7:**
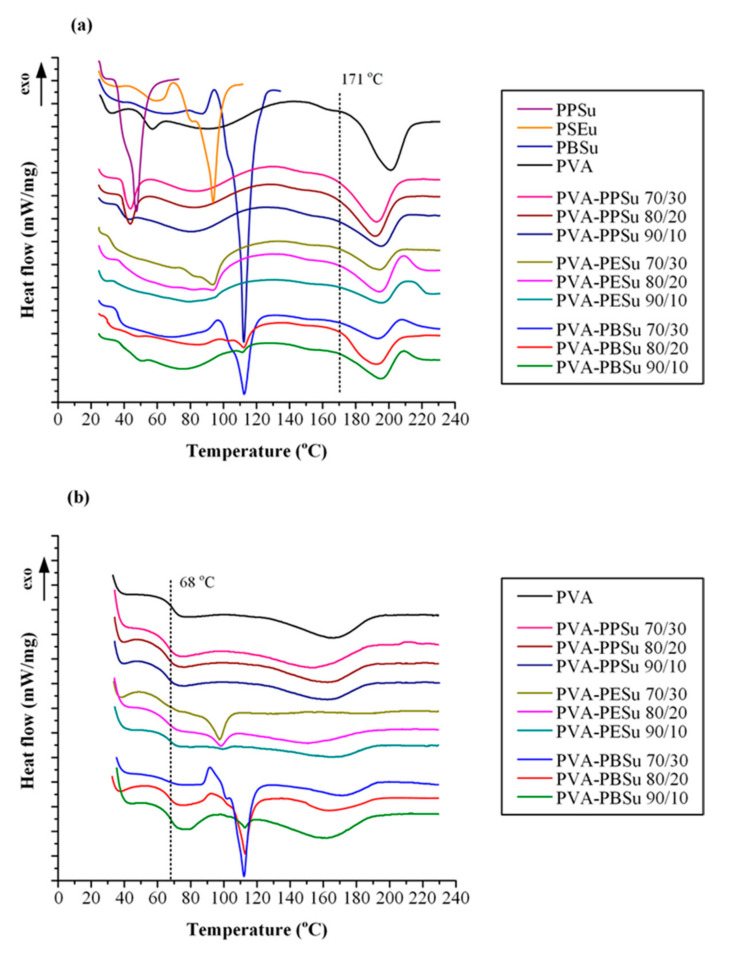
DSC thermograms of the PVA, polyesters and the PVA-polyesters mixtures during (**a**) the 1st and (**b**) the 2nd heating scans.

In addition to thermal stability, the plasticizing effect of polyesters (i.e., the reduction on PVA’s T_g_ and T_m_) was evaluated via DSC. Figure 7a shows the DSC thermograms from the first heating scan of the pure components and the PVA-plasticizer mixtures. In the case of pure PVA, the obtained DSC thermogram showed a single melting endotherm starting at 171.8 °C with ΔH_f_ of 41.69 J/g. In the case of PVA-PPSu mixtures results showed that as the polyester content increases (i.e., from 10 to 30 wt %) PVA’s melting temperature significantly reduces (i.e., from 166 °C to 157 °C, respectively). Similarly, although not so pronounced, the other two poly(alkylene succinate) polyesters also showed a significant reduction in PVA’s melting temperature (30 wt % addition of PESu or PBSu reduced PVA’s melting temperature to 163 °C or 165 °C, respectively).

**Figure 8:**
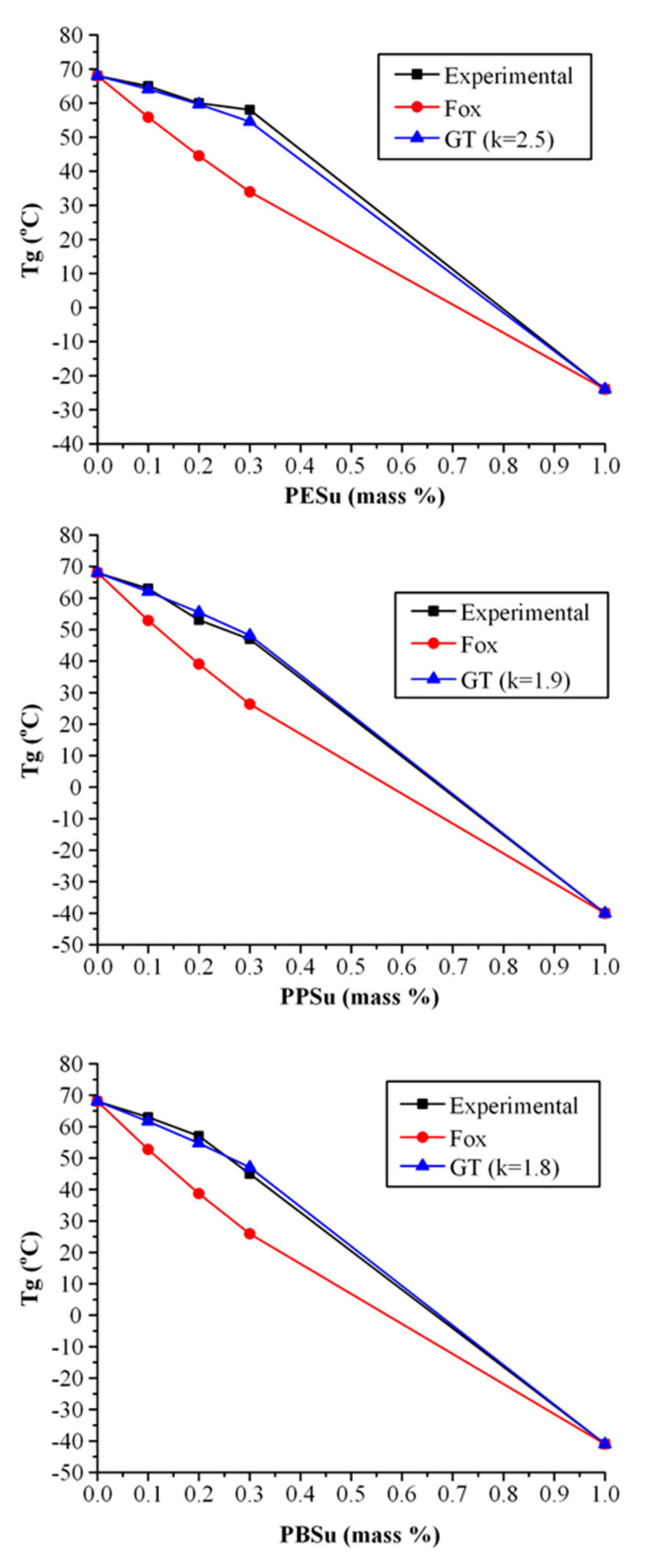
Prediction of T_g_-composition dependence in PVA/polyester blends by using Fox and Gordon Taylor (GT) equations.

Figure 7b shows the DSC thermograms obtained after the melt—quench cooling procedure (i.e., 2nd heating scan). In the case of pure PVA, a single T_g_ was initially recorded at 68 °C, while a broad endothermic peak was also observed at ~170 °C corresponding to PVA’s melting, with the ΔH_f_ of 15.3 J/g indicating that the crystalline part of PVA was significantly reduced after the followed quench cooling procedure. In the case of PVA-polyester mixtures, results showed that except for PPSu (which was amorphous) the rest poly(alkylene succinate) polyesters remained semi-crystalline after quench cooling, with characteristic melting endotherms present at ~95 °C and ~110 °C for PESu and PBSu, respectively. In all cases, increasing polyesters’ content resulted in a significant reduction of T_g_ (compared to the pure PVA) as shown in Figure 8, indicating that all tested poly(alkylene succinate) polyesters may successfully act as plasticizers to PVA.

#### 3.2.3. Physical State Evaluation

**Figure 9:**
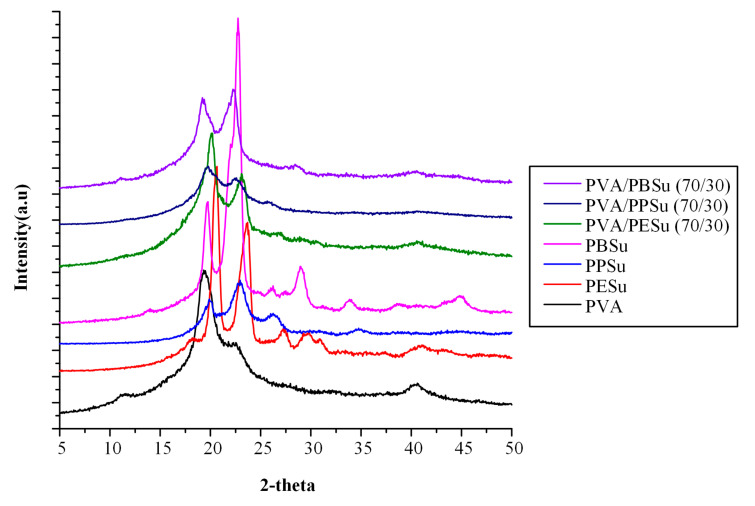
pXRD diffractograms of pure components and PVA-polyesters melt dispersions.

Figure 9 shows the pXRD diffractograms of pure components along with the PVA-polyester melt dispersions. In regard to PVA, pXRD revealed an amorphous halo along with a characteristic strong crystalline reflection at 2θ ~19.5° and a shoulder at ~22.6°, indicating the semi-crystalline nature of the polymer. In regard to the obtained poly(alkylene succinate) polyesters, results from the obtained pXRD diffractograms correspond to the known α crystal forms of these polyesters [48]. Regarding to the PVA-polyester melt dispersions, the obtained patterns showed that all PVA’s characteristic 2θ reflections were present (i.e., at ~19.5° and ~22.6°), revealing that, in all cases, PVA remained semi-crystalline after its processing with the selected low molecular oligomers of poly(alkylene succinate) polyesters. Additionally, except for PPSu, the diffractograms obtained from the melt dispersions showed some of the characteristic crystalline peaks of the polyesters, indicating that, in these cases, both PESu and PBSu remained semi-crystalline within the PVA matrix. Finally, in comparison to the rest polyesters, when PPSu was utilized as a plasticizer a significant reduction in the obtained PVA crystalline reflection peaks was observed, verifying DSC’s findings that in the case of PPSu the crystallinity of the PVA is significantly reduced compared to PESu and PBSu.

#### 3.2.4. Molecular Interactions

In a further step, the formation of molecular interactions between PVA and the examined polyesters was evaluated both experimentally (*via* DSC and ATR-FTIR) and theoretically (*via* MD simulations).

##### DSC Analysis

The formation of molecular interactions between any two miscible compounds (such as a polymer and a plasticizer) may be investigated through DSC, by evaluating the dependence of the T_g_ in regard to components’ mass fractions. Until now, there are several theoretical and empirical equations describing this dependence. Among them, the Fox model expresses this relation with the following equation [49].

[1/T_g_] = w_1_/T_g1_ + w_2_/T_g2_ (7)

where, T_g_ is the glass transition temperature of the polymer-plasticizer blend, w_1_ and w_2_ are the mass fractions of the polymer and the plasticizer that constitute the blend and T_g1_ & T_g2_ are their respective glass transition temperatures.

Accordingly, Gordon and Taylor have proposed a similar equation taking into account the possibility of significant interactions evolving between the studied components [50]:

T_g_ = (w_1_T_g1_+kw_2_T_g2_)/(w_1_+kw_2_) (8)

where, k is a constant representing a semi-quantitative measure of the interaction strength between the reactive groups. If k takes values close to 1 or above then strong interactions between the miscible blend components are taking place [37,51].

Figure 8 shows the experimental T_g_s as well as the theoretical predictions based on the Fox and Gordon-Taylor (GT) approaches. Contrary to the Fox model (where the theoretically estimated T_g_s were not in good agreement with the recorded experimental values) the theoretical predictions for T_g_s according to GT equation, fit quite well to the experimental data, with k parameter being well above one in all cases (i.e., 2.5, 1.9 and 1.8 for PESu, PPSu and PBSu, respectively). This indicates that significant intermolecular interactions are taking place between the two components. However, although the combination of DSC data with theoretical modelling can reveal the presence of molecular interactions, this method does not provide any details regarding to the type of the interaction evolving. Hence, in order to do so, a more suitable method, such as the use of ATR-FTIR spectroscopy is needed.

##### ATR-FTIR Analysis

**Figure 10:**
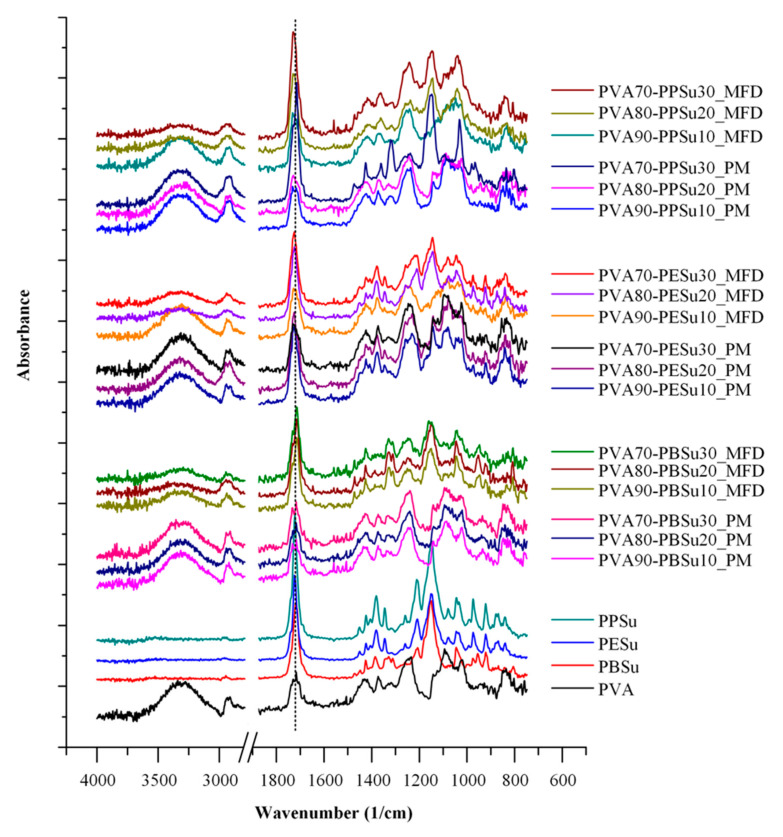
ATR-FTIR spectra of pure components and PVA-polyesters physical mixtures (PMs) and melt-fusion dispersions (MFDs).

Figure 10 shows the ATR-FTIR spectra of pure components, PVA-polyesters physical mixtures (PMs) and melt-fusion dispersions (MFDs) at several ratios. The spectrum of pure PVA showed characteristic peaks at 3301 cm^−1^ (stretching of -OH), 1724 cm^−1^ (stretching of –C=O from the remaining acetyl groups), 1423 cm^−1^ (bending of –OH and wagging of –CH_2_), 1321 cm^−1^ (δ(OH) rocking with CH wagging), 1145 cm^−1^ (shoulder stretching of –CO– from the crystalline part of PVA) and 1083 cm^−1^ (bending of –OH from amorphous part of PVA). Regarding to the neat polyesters, the strong signals at 1717 cm^−1^ and 1155 cm^−1^ could be assigned to the –C=O and –COO– asymmetric stretching vibrations, respectively, which demonstrated the presence of ester linkages. As for the peak at 2960 cm^–1^, it is attributed to the stretching vibration of –CH of the –CH_2_ group. In the case of PVA-polyester mixtures, results showed several differences between the ATR-FTIR spectra of the MFD samples and the corresponding PMs. Specifically, a significant reduction and widening was observed at 3301 cm^−1^, corresponding to the -OH stretching of PVA, while slight shifts (from 1730 cm^−1^ to 1710 cm^−1^) were also observed in the region of 1750–1600 cm^−1^, corresponding to the –C=O and –COOC– stretching of the poly(alkylene succinate) polyesters. These differences indicate that significant molecular interactions (probably hydrogen bonds, HBs) between the hydroxyl hydrogens of PVA and the ester oxygens of the polyesters are being formed during the melt-fusion processing.

##### MD Simulations

In addition to the above experimentally based approaches, an in-depth evaluation of the evolving molecular interactions was attempted with the aid of MD simulations. In general, MD simulations are able to provide detailed atomic-level structural and energetic information that can highly assist the investigation of such molecular interactions.

Figure 4 shows the MD molecular assemblies of the PVA and poly(alkylene succinate) polyester mixtures as resulted after the multistep equilibration protocol employed in the present study. Results showed that in all cases well mixed amorphous structures were constructed. Table 3 summarizes the total interaction energy between polyesters and PVA, estimated by MD simulations according to Equation (6). Results showed that the PVA-PPSu exhibited the lowest interaction energy (−2308 kcal/mol) compared to PVA-PESu (−2201 kcal/mol) and PVA-PBSu (−2274 kcal/mol) mixtures, indicating that PVA-PPSu is the most stable systems. Additionally, this finding explains the complete amorphization of PPSu in the melted PVA samples, since strong interactions are being formed between the two components that restrict the polyester from re-crystallizing.

**Figure 11:**
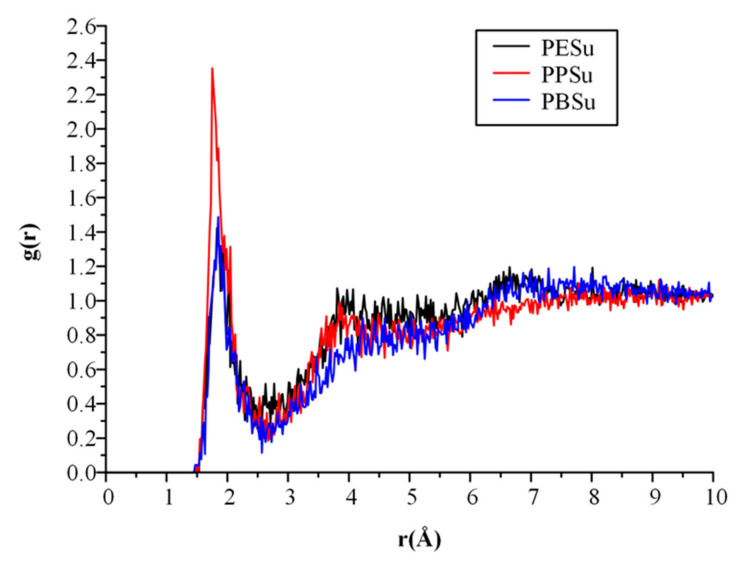
Radial distribution function, *g*(*r*), between PVA’s -OH proton hydrogen donor and polyester’s oxygen acceptors.

In a further step the molecular interactions between the prepared poly(alkylene succinate) polyester and the PVA matrices in the melt state were evaluated by generating the radial distribution functions, *g*(*r*), between the hydrogen atoms of PVA and the oxygen atoms of polyesters (Figure 11). In such diagrams, donor-acceptor distances below 2.5 Å can be characterized as strong interactions, while distances between 2.5–3.2 Å and 3.2–4.0 Å are considered as moderate and weak interactions, respectively. Results showed the formation of a significant *g*(*r*) peak at 2.09 Å, 2.00 Å and 2.08 Å for PVA-PESu, PVA-PPSu and PVA-PBSu, respectively, indicating the presence of strong HBs between the hydroxyl hydrogens of PVA and the ester oxygens of all poly(alkylene succinate) polyesters. In addition, the obtained *g*(*r*) results showed that PPSu forms slightly stronger HBs with PVA, since the peak for PPSu is slightly higher and located in a shorter r distance than the rest polyesters.

#### 3.2.5. Melt Flow Properties

**Figure 12:**
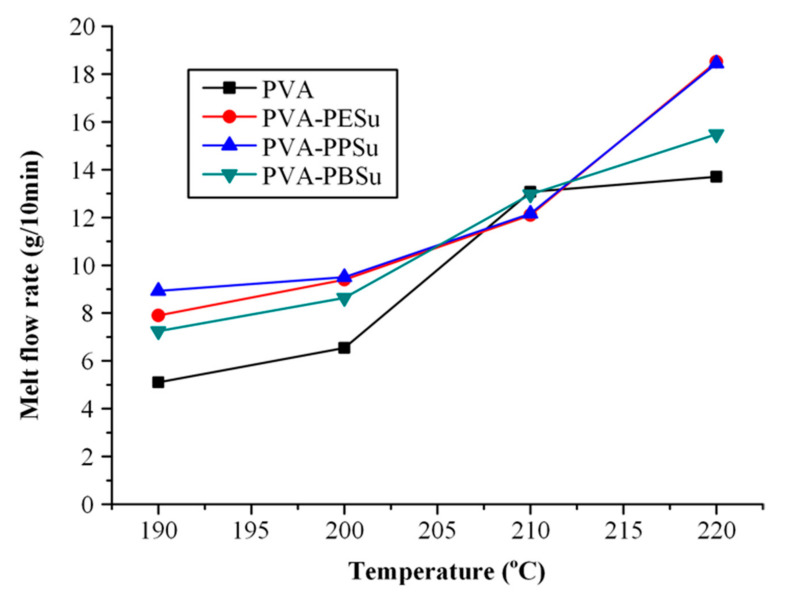
MFI vs. temperature for pure PVA and the PVA-polyesters mixtures.

In the final step of the present study, the effect of the selected polyesters on the melt flow properties of PVA was evaluated by measuring the MFI. In general, measurement of MFI is a common analytical method which is used as an indicator of the polymer’s viscosity and the flow properties of the polymer melt at a certain temperature and applied pressure. Particularly, MFI is defined as the mass of the polymer extruded in ten minutes through an orifice of specific dimensions. The dimensions of the orifice, as well as the mass load and the temperature conditions are specified by ASTM D 1238 [52]. Although MFI is considered as the simplest, standardized measurement of the flowability of a polymer melt, it is not a fundamental polymer property. It is an empirically defined parameter critically influenced by the conditions of the measurement [53,54]. Nevertheless, it is suitable for quality control or comparative studies and it is still considered by the industry as a relatively cost-effective and quick analytical method to indicate the polymer’s flow properties. Figure 12 shows the MFIs of pure PVA and PVA mixtures with 30 wt %. PBSu, PESu and PPSu. Results showed that in all tested temperatures (with the exception of 210 °C) the addition of plasticizers resulted in higher MFIs, indicating better melt flowability, while a comparison of the MFIs for all PVA-polyesters mixtures, showed that PESu and PPSu had the greatest impact in terms of melt flow improvement.

## 4. Conclusions

The thermal degradation of PVA (which takes place almost immediately after its melting) as well as its significant processability problems at temperatures below its melting point, makes PVA’s thermal treatment in pharmaceutical applications extremely difficult. Taking this limitation into consideration, in the present study three low molecular weight oligomers of poly(alkylene succinate) polyesters were synthesized and their efficacy as plasticizers for PVA fusion-based pharmaceutical applications was evaluated. According to the obtained results, all synthesized polyesters were miscible with PVA, while only PBSu and PPSu resulted in an acceptable thermal stability profile. It is important to note that the miscibility of the components was successfully predicted via MD simulations and was experimentally verified through HSM analysis. In addition, by using PPSu and PESu as plasticizers PVA’s melt flow properties were improved. Afterwards, the utilization of ATR-FTIR spectroscopy revealed the formation of significant molecular interactions during melting in all tested systems, while the results obtained from the radial distribution function estimations showed that PPSu forms slightly stronger HBs with PVA. In conclusion, although a deeper insight into the prepared systems is still needed, the results of the present study indicate that PPSu may be a suitable plasticizer for reducing PVA’s processing temperature without affecting its thermal decomposition profile.

## Figures and Tables

**Table 1:** Intrinsic viscosity, molecular weights and thermal properties of the prepared poly(alkylene succinate) polyesters (PESu, PPSu and PBSu).

Polyester	[η] (dL/g)	Mn (g/mol)	Mw (g/mol)	Mw/Mn	T_m_ (^o^C)	T_g_ (^o^C)	T_c_ (^o^C)	ΔH (J/g)	CF_c_ (%)
PESu	0.13	1196	3056	2.56	93	−24	27	68	40
PPSu	0.07	2823	6551	2.36	45	−40	-	-	-
PBSu	0.16	4238	8833	2.08	111	−41	77	85	5

**Table 2:** HSPs, MD-based solubility parameters (*δ_MD_*) and the absolute difference between the corresponding values (Δ*δ_t_*) for PVA and the prepared poly(alkylene succinate) polyesters (PESu, PPSu and PBSu).

	Substances			
	PVA	PESu	PPSu	PBSu
HVK group contribution method				
HSPs (MPa^1/2^)	34.0	21.6	21.9	22.1
Δδ_t_ (MP^1/2^)	-	12.4	12.1	11.9
MD simulations				
δ_MD_ (MPa^1/2^)	26.1	26.8	25.5	24.4
Δδ_t_ (MP^1/2^)	-	0.7	0.6	1.7

**Table 3:** Interaction energy (*E_inter_*) of PVA-polyesters estimated via MD simulations.

PVA-Plasticizer	E_inter_ (kcal/mol)
PVA-PESu	−2201
PVA-PPSu	−2308
PVA-PBSu	−2274

## Data Availability

Data is contained within the article.
